# A quality improvement project to increase breast milk feeding of hospitalized late preterm infants in China

**DOI:** 10.1186/s13006-023-00582-0

**Published:** 2023-08-23

**Authors:** Meiying Quan, Zhenghong Li, Laura Placke Ward, Shuju Feng, Yalin Jing, Lin Wang, Jing Yuan

**Affiliations:** 1 Pediatric department, State Key Laboratory of Complex Severe and Rare Disease, Peking Union Medical College Hospital, Chinese Academy of Medical Science and Peking Union Medical College, No.1 Shuaifuyuan, Wangfujing, Dongcheng District, Beijing, 100730 China; 2 Division of Neonatology, NICU, Cincinnati Children’s Hospital Medical Center, Ohio, United States

**Keywords:** Quality improvement, Breastfeeding, Late preterm infant

## Abstract

**Background:**

The breastfeeding rates of late preterm infants are lower than both term and extremely preterm infants. To explore the interventions of increasing full breast milk feeding rate of hospitalized late preterm infants on the 7th day after birth (D7) and evaluate the effect of these quality improvement (QI) interventions.

**Methods:**

The full breast milk feeding (amount of enteral breast milk reached 120ml/kg/d on D7) rate of hospitalized late preterm infants during May 2017 and November 2017 was set as the baseline before intervention, and the specific aim of promoting breast milk feeding was put forward. The Pareto Chart was used to analyze the factors that affect breast milk feeding process, as well as the discussion of multidisciplinary experts. Key drivers were constructed, including informational materials and education about breast milk feeding, consultations and support on optimal breast milk initiation, initiating breast milk expression within one hour after birth, accurate measurement and recording of expressed breast milk, stimulating continuous and effective lactation, proper breast pump selection in and out of hospital and sending and preserving of expressed milk to NICU. Control chart was used to monitor the monthly change of full breast milk feeding rate until the aim was achieved and sustained.

**Results:**

The baseline of full breast milk feeding rate of late preterm infants was 10%, and the aim of QI was to increase the rate to 60% within a two-year period. Control chart dynamically showed the full breast milk feeding rate increased to 80% with the implementation of the interventions, achieved and made the aim of QI sustained.

**Conclusion:**

QI interventions including breast milk feeding education, early postpartum breast milk pumping, kangaroo care to stimulate breast milk secretion, and convenient way of transporting breast milk to NICU, could significantly improve the full breast milk feeding rate of hospitalized late preterm infants.

**Supplementary Information:**

The online version contains supplementary material available at 10.1186/s13006-023-00582-0.

## Background

Breast milk is the best enteral nutrition choice for infants, advantages of breastfeeding has been emphasized extensively. Clinical investigation demonstrated that the exclusive breastfeeding rate of term infants could reach 87.3% at discharge in Australia [1]. Breast milk has been the preferred enteral feeding option for extremely low and very low birth weight infants (infants with birth weight less than 1000 and 1500 g respectively), because of the protective function against infection and necrotizing enterocolitis (NEC) [2]. The exclusive breast milk feeding rate in certain neonatal intensive care units (NICU) in China is as high as 80% for extremely preterm infants [3]. However, for late preterm infants, those born between 34 weeks and 36^+ 6^ weeks, breast milk feeding rates are much lower, especially those transferred to NICU for further medical care. Late preterm infants are not as mature as term infants who could effectively initiate and maintain lactation, and yet not as fragile as extremely preterm infants who are reliant on the biological benefits of breast milk [4]. Breast milk feeding was not paid enough attention among this population. Data from Beijing demonstrated that the exclusive breast milk feeding rate (breast milk feeding from birth, not given any formula) for hospitalized late preterm infants was only 4.5% [5]. Improving breast milk feeding during hospitalization and maintaining breast milk feeding after discharge remains challenging in China [6]. Mothers of late preterm infants who intend to breast feed may not express enough milk on the first few days following delivery, and in some medical centers, the sending and preserving of breast milk to the NICU is another obstacle. Nevertheless, late preterm infants are the largest population among preterm infants, accounting for 74% of all preterm infants [7]. Changes in practice targeting these infants are likely to have a major impact on health outcomes and medical care resources [8, 9].

Studies on quality improvement (QI) initiatives and methodology have been extensively published and play an important role in medical quality improvement [10–13]. The Vermont Oxford network and Canada neonatal network are QI databases for preterm infants in the United States and Canada respectively. A similar network for late preterm infants has been established for years in Beijing. Full breast milk feeding rate of hospitalized late preterm infants was not satisfactory, so we planned to track the change while implementing evidence-based QI interventions. The aim was to increase the full breast milk feeding rate by at least 50% over a two-year period.

## Methods

### Context

This is a single center QI study at Peking Union Medical College Hospital (PUMCH) in Beijing, China. PUMCH is a university-affiliated general hospital, with approximately 3500 annual deliveries, and a 25-bed NICU with approximately 800–900 annual admissions. Our team takes care of all the infants born in PUMCH. A neonatologist rounds in the obstetrics department and cares for neonates rooming in with their mothers. Breast pumps are available in maternity ward. A portion of late preterm infants, mostly were 34 and 35 weeks of gestation, will be transferred to NICU for further medical care and separated from their mothers. The NICU is equipped with refrigerators to store breast milk and heaters to reheat breast milk.

### Study team

The QI team included neonatologists and nurses from obstetric units and NICU. In addition, social workers were invited to participate the QI project.

### Study population

Late preterm infants born at PUMCH and admitted to the NICU on the first day of life during May 2017 and November 2019 were qualified for this QI study. Infants with gastrointestinal anomalies, severe infection, and congenital metabolic disease were excluded. In addition, infants were excluded if the length of hospital stay was less than 7 days, or if their mothers did not intend to breast feed the infants.

### Measures

The primary outcome measure was the rate of full breast milk feeding, which we defined as the percentage of late preterm infants who reached 120ml/kg/d of enteral breast milk feeding on the 7th day (D7). This was based on previous data that the median length of hospital-stay for NICU admitted late preterm infants was 8 days (7-11days), and enteral feeding at discharge was 108.7ml/kg/d [8]. We speculated that the minimum volume of breast milk to maintain full breast milk feeding would be 120 ml/kg/d by discharge. So we defined ***full breast milk feeding*** as reaching 120ml/kg/d of breast milk on D7. On the control chart, late preterm infants were easily and clearly grouped by five to calculate full breast milk feeding rate. In addition, data were reviewed monthly for potential errors. We defined our SMART (specific, measurable, attainable, relevant, time-based) aim as 50% increase of full breast milk feeding rate for hospitalized late preterm infants by November 2019.

### Interventions

**Fig. 1:**
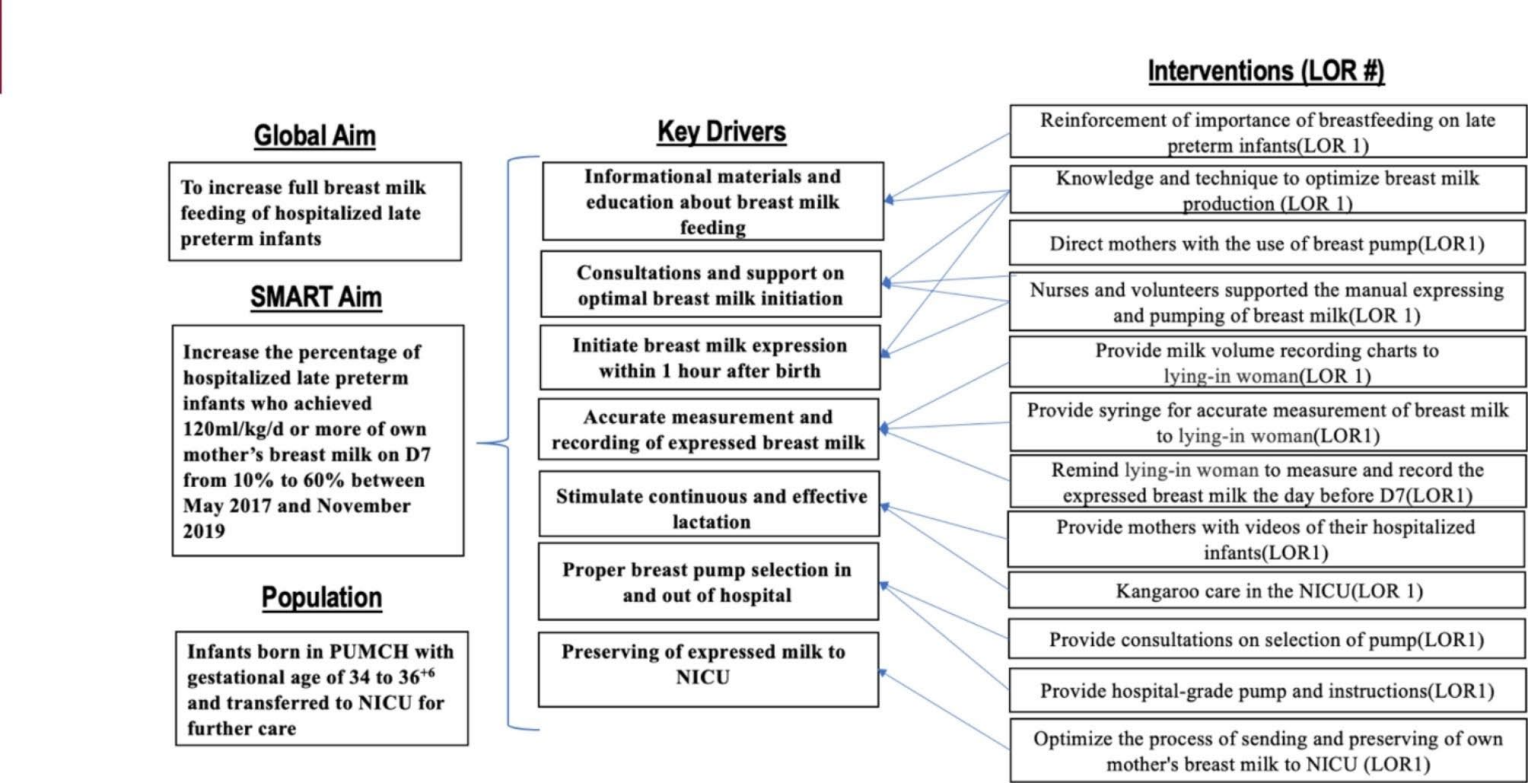
Key driver diagram to increase full breast milk feeding of late preterm infants. Note: LOR # = Levels of Reliability Number, e.g., LOR 1; SMART: specific, measurable, attainable, relevant, time-based; NICU neonatal intensive careunit;

Active QI efforts began in December of 2017 and completed in November of 2019. Multidisciplinary team mapped current processes based on the best evidence from the literature, experience, previous data and failure mode and effect analyses. Developed key drivers to prioritize QI interventions, including informational materials and education about breast milk feeding, consultations and support on optimal breast milk initiation, initiating breast milk expression within 1 h after birth, accurate measurement and recording of expressed breast milk, stimulating continuous and effective lactation, proper breast pump selection in and out of hospital and sending and preserving of expressed milk to NICU (Fig. 1). Specific interventions were identified and tested with Plan Do Study Action (PDSA) cycles (Table 1). Outcome and processes data were shared at monthly meetings.

### Early initiation of milk expression

The timing of breast milk pumping initiation after giving birth to very low birth weight infants has been studied extensively. In a randomized study, mothers who used a hospital-grade electric breast pump within the first hour post-delivery produced significantly greater cumulative human milk output at Day 7 and Week 3 compared to mothers who used the same breast pump after the first hour post- delivery [15, 16].

The nurses in maternity ward and social workers assisted mothers separated from their late preterm infants in initiating milk expressing as soon as possible after birth, mostly within one hour, to achieve 8 to 12 times of milk expressing per day. Both manual and pump expressing are effective in establish and maintain lactation.

**Table 1:** Interventions and starting time for each intervention

Category of intervention	Specific interventions	Starting time
**Informational materials and education about breast milk feeding**	1. Reinforcement of importance of breastfeeding on late preterm infants	2017-12-12
	2. Knowledge and technique to optimize breast milk production	2018-4-28
**Consultations and support on optimal breast milk initiation**	1. Knowledge and technique to optimize breast milk production	2018-4-28
	2. Direct mothers with the use of breast pump	2017-12-12
	3. Nurses and volunteers supported the manual expressing and pumping of breast milk	2018-5-12
**Initiate breast milk expression within 1 h after birth**	1. Knowledge and technique to optimize breast milk production	2018-4-28
	2. Nurses and volunteers supported the manual express and pumping of breast milk	2018-5-12
**Accurate measurement and recording of expressed breast milk**	1. Provide milk volume recording charts to lying-in woman	2017-12-12
	2. Provide syringe for accurate measurement of breast milk to lying-in woman	2017-12-27
	3. Remind lying-in woman to measure and record the expressed breast milk the day before D7	2018-1-30
**Stimulate continuous and effective lactation**	1. Provide mothers with videos of their hospitalized infants	2018-5-12
	2. Kangaroo care in the NICU	2018-4-12
**Proper breast pump selection in and out of hospital**	1. Provide consultations on selection of pump	2018-7-20
	2. Provide hospital-grade pump and instructions	2018-11-16
**Optimize sending and preserving of expressed milk to NICU**	Optimize the process of sending and preserving of own mothter’s breast milk to NICU	2019-2-11

### Optimal devices for milk pumping

Hospital grade breast pumps could mimic the infant’s sucking rate, rhythm and pressures to the greatest possible extent in pump-dependent women. Studies have proved the effectiveness, efficiency, comfort and convenience of breast pumps and breast pump suction patterns compared with the infant as the “gold standard” [17, 18]. New hospital grade breast pumps were obtainable in the maternity ward.

### Lactation stimulation

Suckling by the newborn helps to maintain lactation by the release of oxytocin, which causes contraction of the mammary epithelium and the ejection of milk [19]. When mothers and infants are separated, there is interruption of this suckling. Kangaroo care may partially compensate for this condition. Kangaroo care is a cost-effective intervention recommended by the World Health Organization for the care of stable preterm infants. Infants may receive skin-to-skin care on the chest of their mother or another caregiver while exclusively breastfeeding (ideally) or while receiving breast milk feedings. Data showed that intermittent kangaroo care was associated with a nearly doubled increase in exclusive breast milk feeding and breastfeeding at both discharge and 42 days after discharge for late preterm infants [20–22].

To promote kangaroo care in NICU, we provided comfortable reclining chairs for mothers and hand-held mirrors to observe their infants while performing kangaroo care. The length of time that mothers could stay in the NICU with their infants was increased to at least two hours per day. Videos and photos of hospitalized infants were sent to the smartphone of parents to increase emotional connections and further stimulate lactation.

### Sending breast milk to the NICU

Parents were encouraged to send expressed breast milk to NICU at any time. Refrigerators for storage of breast milk and milk heaters were equipped in the NICU.

Sending and storage of breast milk was standardized in the NICU as well as the key points was demonstrated to the parents.

### Analysis

For the primary outcome, baseline median and control limits were calculated and displayed from May 2017 through November 2017. The baseline mean was carried forward and displayed throughout the intervention period from December 2017 to November 2019. Data values were added monthly and monitored for evidence of significant change by using standard SPC rules, including the presence of (1) 1 point outside the upper or lower control limits, (2) 2 of 3 successive points in the outer third of the control limit, (3) 8 successive points above or below the center line, or (4) 6 consecutive points increasing or decreasing [10].

## Results

### Baseline data

**Fig. 2:**
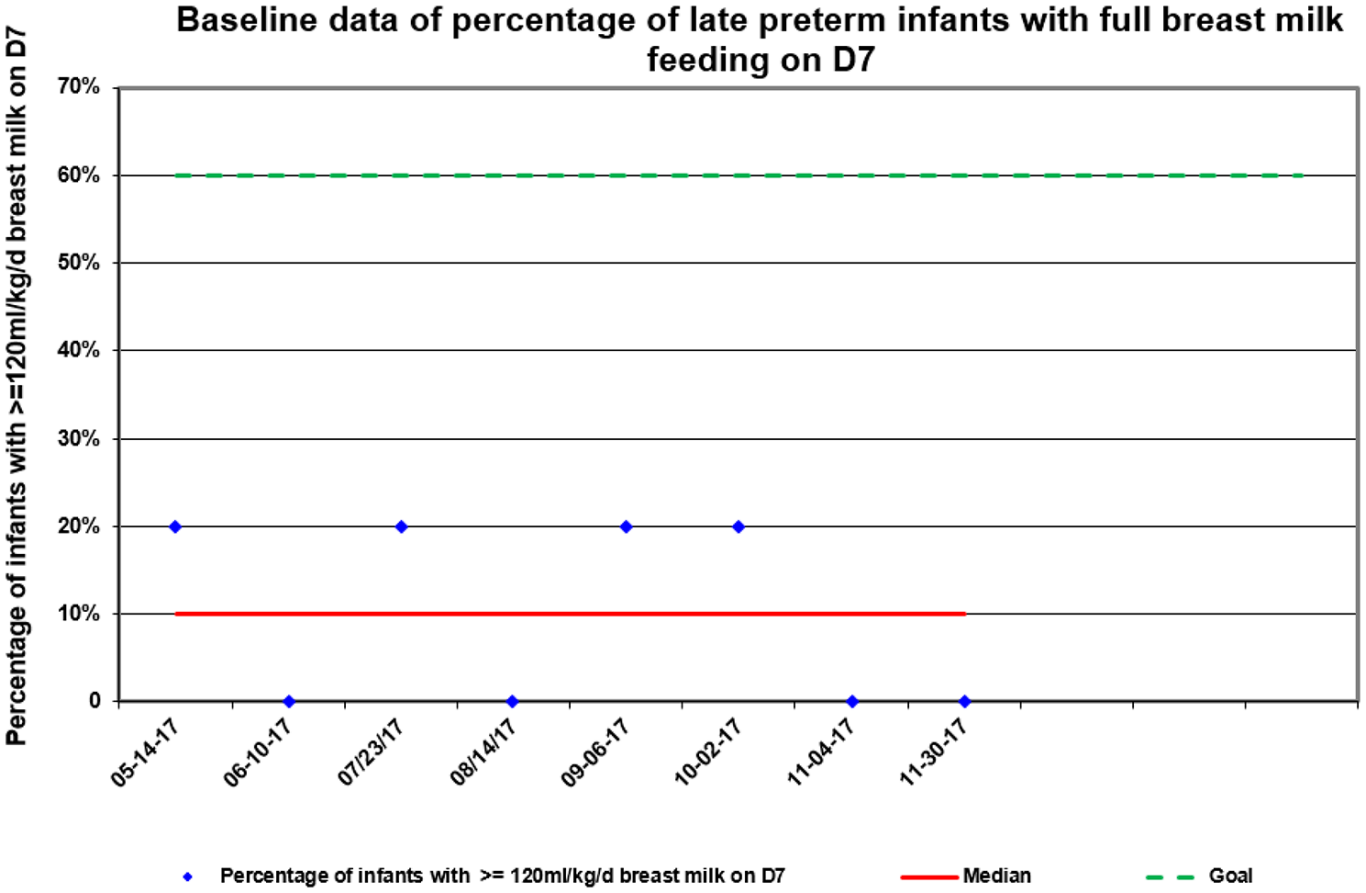
Baseline data of full breast milk feeding rate on D7

We determined the baseline full breast milk feeding rate in 40 late preterm infants that qualified between May 2017 and November 2017.These infants were grouped by 5, and the average full breast milk feeding rate was 10% (Fig. 2).

There were about two to four late preterm infants who were not included in this QI project at each time point despite five eligible infants because major abnormality, hospitalized less than seven days or whose mother refused to breast feed.

### Pareto of failures analysis

**Fig. 3:**
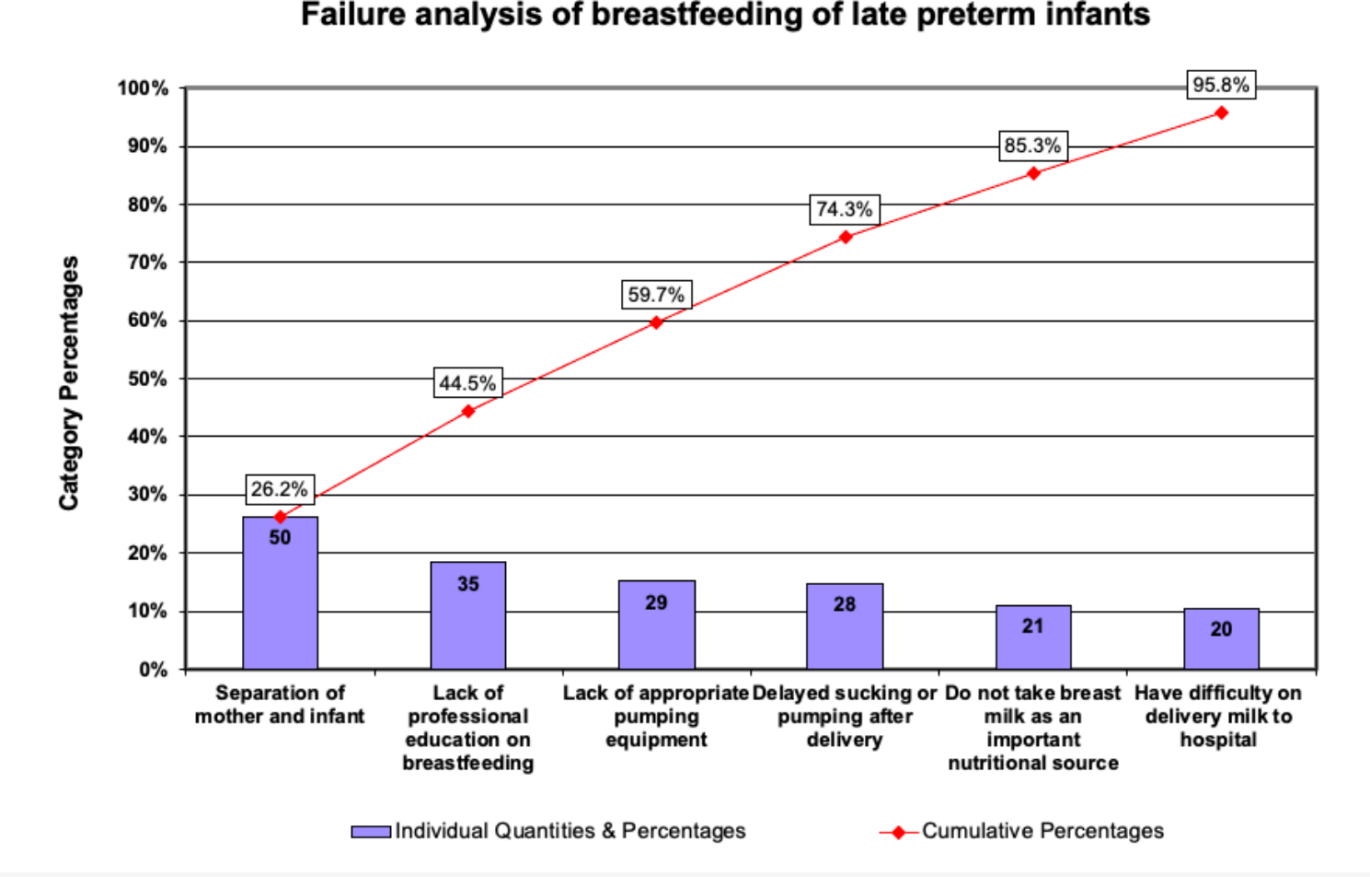
Pareto analysis of failure to breast feed hospitalized late preterm infants

The reasons of failure to achieve full breast milk feeding were analyzed through telephone inquiries and questionnaires to 191 mothers of late preterm infants during the year of 2017. Pareto of failures analysis illustrates the most common factors fail to achieve full breast milk feedings and include (A) separation of mother and infant. (B) lack of professional instruction on breastfeeding of late preterm infants. (C) lack of appropriate pumping equipment. (D) delayed sucking or pumping after delivery. (E) Do not take breast milk as an important enteral nutritional source. These five reasons accounted for 85.3% of failures of breast milk feeding (Fig. 3).

**Fig. 4:**
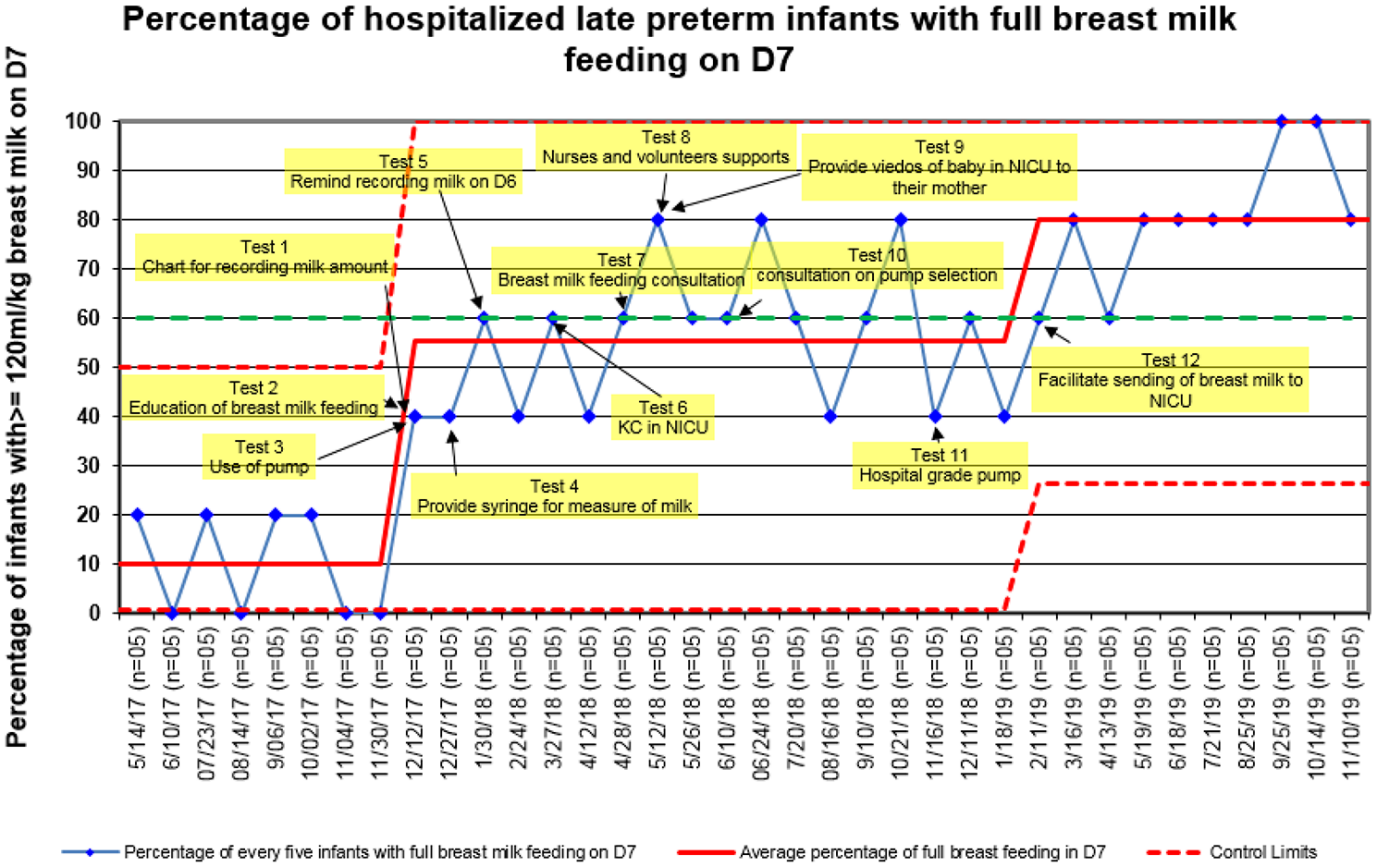
Control chart of full breast milk feeding rate on the 7th day of life. Note: the blue dot on the control chart was calculated every five late preterm infants as the percentage of infants reaching full breast milk feeding on D7. The data was displayed throughout the intervention period from May 2017 to November 2019. KC: Kangaroo care

A total of 140 eligible late preterm infants were analyzed during December 2017 and November 2019 on the control chart with 28 infants excluded because of major abnormality, the length of hospital stay was less than 7 days or their mothers did not intend to breast feed. The primary outcome measure, full breast milk feeding rate on D7, increased from 10 to 80% (Fig. 4) and sustained on this level by project completion.

## Discussion

The benefits of breast milk are well documented, and exclusive breastfeeding is recommended for all infants for the first 6 months of life. The advantages conferred by breast milk feedings are especially important in very low and extremely low birthweight infants due to the nutrients in breast milk, as well as the protective effects against infection and inflammation. However, these advantages are equally important in the vulnerable late preterm group [23, 24]. Our hospital supports a breast milk bank to augment the breast milk supply of very low and extremely low birth weight infants who cannot get enough own mother’s milk in the early stage of life. But most late preterm infants do not qualify for donor milk feeding and can only rely on their own mother’s milk, or alternatively, formula. Breast milk feeding of late preterm infants presents several challenges. Late preterm infants may be sleepier and have reduced muscle strength and therefore have more difficulty with latch, sucking and swallowing [14]. Delayed lactogenesis also contributes to difficulties with breastfeeding initiation. When mothers and infants are separated, the amount of own mother’s breast milk may fail to meet the needs of late preterm infants. How to improve full breast milk feeding rate of hospitalized late preterm infants and sustain breast milk feeding after discharge? There is a paucity of literature examining the factors affecting breast milk feeding practices of hospitalized late preterm infants. We conducted this QI project to explore effective interventions to increase the full breast milk feeding rate of hospitalized late preterm infants.

Daily feeding amount of 120ml/kg and 150ml/kg are chosen as full enteral feeding standard for preterm infants, and 120ml/kg/d is the minimum requirement for late preterm infants in the early stage of life [24]. Compared with very low and extremely low birth weight infants, late preterm infant appears to be more mature on digestive ability. Therefore, it is more likely that they will sustain full enteral feeding after discharge if they could tolerate 120ml/kg/d of breast milk in hospital. Our previous study showed that, the average length of hospital stay was 7 to 11 days in this population [8]. Thus, we defined breast milk feeding of 120ml/kg/d or more on the D7 as full breast milk feeding. We choose this index as the primary outcome measure of this QI project, and grouped every five late preterm infants to clearly plot the percentage of infants reaching full breast milk feeding on the control chart.

This project introduced multiple interventions. First, we focused on the importance of breast milk feeding on late preterm infants. Substantial efforts have been placed on the importance of own mother’s breast milk for very low and extremely low birth weight infants, and this experience can be drawn as a reference for late preterm infants. Both the medical care givers and parents should recognize the importance of breast milk feeding and its irreplaceable impact for late preterm infants, this “near term” population [5]. In terms of specific measures, we set up a multidisciplinary breast milk feeding steering team, familiar with the knowledge and skills of assisting breastfeeding initiation and maintaining lactation. This team was consisted with nurses, obstetricians and neonatologists, as well as social workers. The importance of breast milk feeding was emphasized to expectant parents at multiple points, including prenatal visits, postpartum period, and on admission to the NICU. Except for lecture in pregnant school and breast milk feeding posters in clinic, multi-media brochures and short videos containing key points of breast milk feeding were spread to the expectant parents by WeChat. Lactation consultation was available during hospital stay. In addition to the technical support of breast milk feeding, tools for measuring and recording the volume of breast milk was also provided to mothers of hospitalized late preterm infants. Accurate measurements and recording of expressed breast milk could display the continuous increments of postpartum breast milk production and therefore promote mother’s confidence in breast milk feeding.

Frequent and ongoing lactation stimulation can help the mothers with a more positive breast milk feeding outcome and may compensate for the maternal/infant separation and the interrupted sucking connection. Therefore, we supported new mothers to initiate milk expression within one hour after delivery. In our hospital, the hospital-grade pumps were available in maternity ward. Data from randomized controlled trials have demonstrated that hospital-grade electric breast pumps which were installed with a biphasic suction mode were similar to the unique sucking mode of infants and can enable the mothers to extract milk more efficiently by day of life 14 [18, 20]. The skin-to-skin contact that occurs with breast milk feeding can provide additional advantages. Literature focusing on kangaroo care in the NICU demonstrated its benefits on improvement of exclusive breast milk feeding rate of late preterm infants at discharge and at 42 days after discharge [22]. Kangaroo care has a history of more than 30 years since it was first proposed and applied clinically in 1983. We encouraged mothers to participate kangaroo care as soon as possible when their postpartum physical conditions permitted and provided them with audiovisual materials of late preterm infants taken in the NICU. These interventions aimed to stimulate lactation have become an important part of the systematic management of late preterm infants in our NICU. It has played a very important role in increasing the production of own mother’s breast milk and brought no more risk of nosocomial infection.

Regular and accurate data monitoring is essential for any QI project. The distinction of QI study from traditional research is the continuous tracking and demonstration of outcome data which superimposed interventions during the QI project. Statistical analysis can be visualized throughout the QI process instead of after the completion of the study [11, 12]. The PDSA cycles of each intervention also helped the team to determine the impact of the interventions and whether to be included in the final effective intervention measures. The methodology of QI enabled us to analyze the potential problems behind the data.

This study is a single center QI study. We determined a series of effective interventions, such as emphasizing the importance of breast milk feeding, early milk expression, and interventions stimulating lactation could promote breast milk feeding of hospitalized late preterm infants. Some interventions, such as the multi-dimensional education of breast milk feeding, aggressive lactation guidance and stimulation, and facilitating the sending of breast milk to NICU, could be replicated in other centers. However, some interventions may not be generalizable to all medical institutions, such as kangaroo care of late preterm infants, the application of hospital-grade breast pumps and assistance of social workers. Different medical centers could map out local aims according to unique medical circumstances and take effort to carry out targeted QI projects. We believe that with further development of the preterm database and flexible application of QI methodology, medical care quality and prognosis of late preterm infants will be further improved.

### Electronic supplementary material

Below is the link to the electronic supplementary material.

Supplementary Material 1

## Data Availability

The datasets used and/or analyzed during the current study are available from the corresponding author on reasonable request.

## Abbreviations

QI: quality improvement
NEC: necrotizing enterocolitis
NICU: neonatal intensive care units
PUMCH: Peking Union Medical College Hospital
PDSA: Plan Do Study Action
