# Supplementary material for: A quality improvement project to increase breast milk feeding of hospitalized late preterm infants in China
Source: Int Breastfeed J. 2023 Aug 23;18:45. doi: 10.1186/s13006-023-00582-0 (PMC10463707; doi:10.1186/s13006-023-00582-0)
Supplement: Supplementary file 1 — Supplementary Material 1 [file 13006_2023_582_MOESM1_ESM.docx]

**Examples of breast milk feeding education in WeChat messaging service**


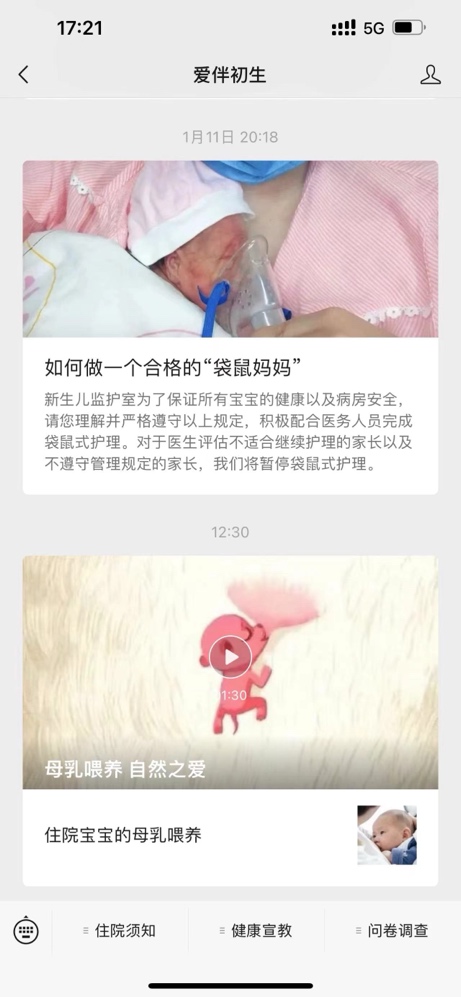

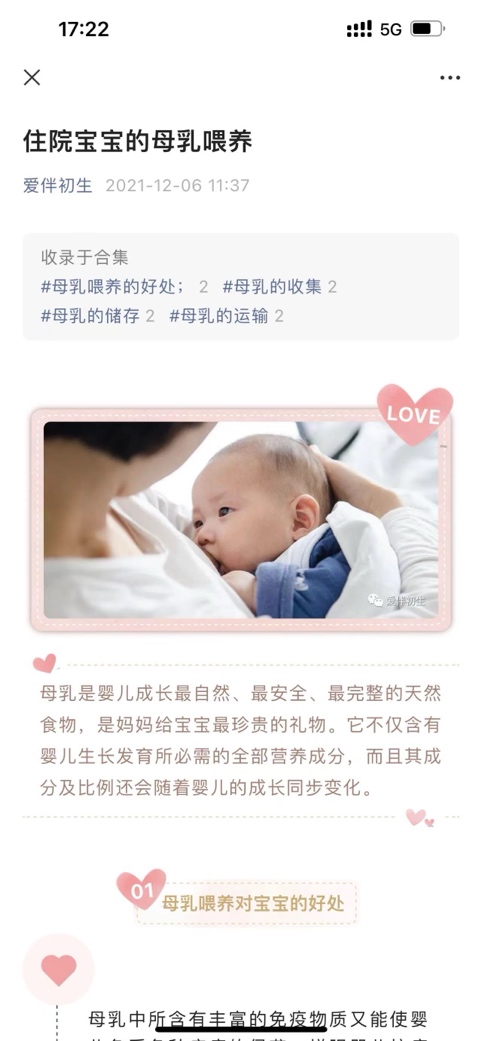


**Breast milk feeding of hospitalized infants**

**How to be a good kangroo mother**


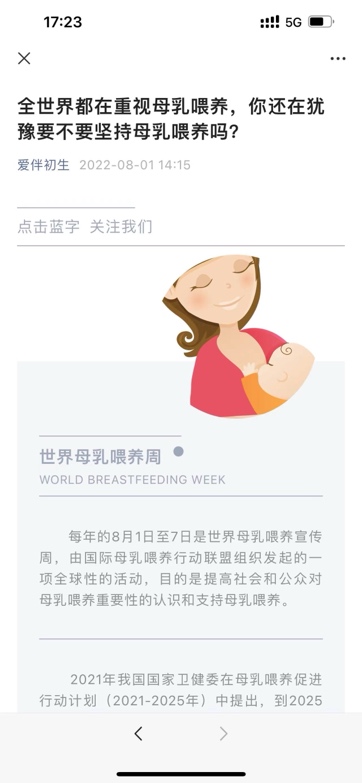

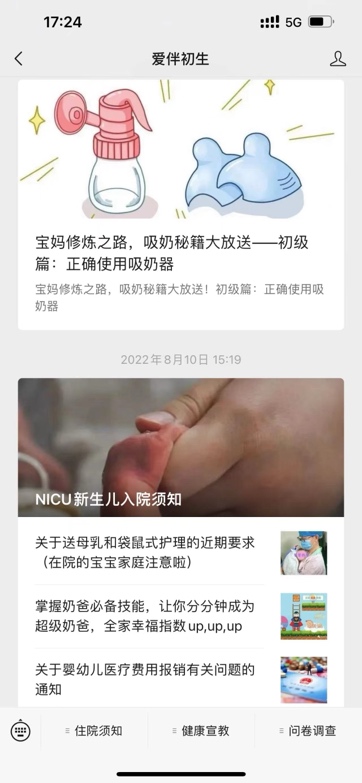

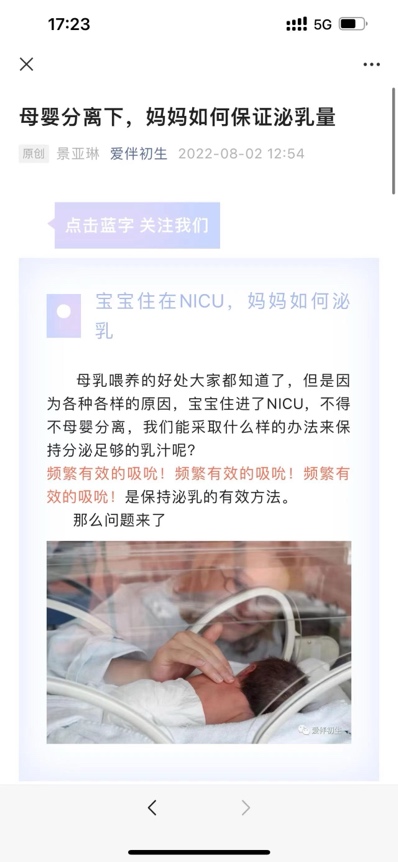


**How to use pump**

**Breast milk feeding, natural love！**

**How to ensure breast milk production when separated with babies**

**Do not hesitate to breastfeed**
